# Evaluation of an automated antimicrobial susceptibility testing system performance for colistin susceptibility in carbapenem-resistant *Acinetobacter baumannii* isolates

**DOI:** 10.1128/spectrum.00859-25

**Published:** 2025-09-30

**Authors:** Arzu İlki, Fatih Mehmet Akıllı, Sevim Alpsoy Özsoy, Zeynep Saygı, İsmail Yüceel-Timur

**Affiliations:** 1 Department of Medical Microbiology, Marmara University School of Medicine, Istanbul, Turkey; 2 Marmara University Pendik Research and Training Hospital, Microbiology Laboratory, Istanbul, Turkey; 3 Sincan Training and Research Hospital, Microbiology Laboratory, Ankara, Turkey; 4 Marmara University School of Medicine, Istanbul, Turkey; 5 Global Medical Affairs, bioMérieux, Marcy l’Étoile, France; Institut National de Santé Publique du Québec, Sainte-Anne-de-Bellevue, Québec, Canada

**Keywords:** carbapenem-resistant *Acinetobacter baumannii*, colistin resistance, VITEK 2 AST-XN21, broth microdilution, *mcr *genes, antimicrobial susceptibility testing

## Abstract

This study aims to assess the efficacy of VITEK 2 AST-XN21 (bioMérieux, Marcy l’Étoile, France) cards in identifying colistin susceptibility, comparing their performance with broth microdilution (BMD) results. A total of 187 carbapenem-resistant *Acinetobacter baumannii* were included and identified by MALDI-TOF (VITEK MS, bioMérieux, Marcy l’Étoile, France), and colistin susceptibility was determined by BMD and the VITEK 2 AST-XN21 card. The cs02n version is used in the VITEK 2. Isolates were evaluated for essential (EA) and categorical (CA) agreements, error rates, and ISO 20776-2:2021 bias rate (acceptable if bias rate is between −30% ≤ bias ≤+30%). Minimum inhibitory concentration (MIC) was interpreted according to the European Committee on Antimicrobial Susceptibility Testing breakpoints (susceptible ≤2 µg/mL; resistant >2 µg/mL). All isolates were subjected to in-house multiplex PCR for possible resistance genes *mcr*-1, *mcr*-2, *mcr-*3*, mcr-*4*,* and *mcr-*5. Out of 187 isolates, 16.04% (*n* = 30) of all isolates were determined as colistin resistant by the BMD method. MIC_50_ and MIC_90_ of the isolates were detected as 1 and 4 mg/L, respectively. When VITEK 2 AST-XN21 results were compared with BMD, CA and EA were 94.6% (177/187) and 89.1% (156/175), respectively. Very major error (3.2%, 6/187) and major error (2.1%, 4/187) were detected. ISO 20776-2:2021 bias rate was 3.6%. No *mcr*1-5 genes were detected.

**IMPORTANCE:**

Colistin resistance in carbapenem-resistant *Acinetobacter baumannii* (CR-Ab) remains a global problem, and broth microdilution constitutes the workload of routine clinical microbiology laboratories. This study is the first to evaluate the cs02n version in the VITEK 2. Our results suggest that VITEK 2 AST-XN21 cards could be an alternative method to detect colistin resistance in CR-Ab isolates.

## INTRODUCTION

Carbapenem-resistant *Acinetobacter baumannii* (CR-Ab) is in the critical group of World Health Organization priority pathogens according to the 2024 list (1). A significant increase has been observed in the number of cases of CR-Ab reported from many countries worldwide (2). Infections caused by CR-Ab strains are very difficult to treat and result in high mortality (2). Unnecessary and inappropriate use of antibiotics in the treatment of infections caused by gram-negative bacteria in hospitals is among the most important reasons for the increase in multi-resistant bacteria (3). In its 2019 report, the Centers for Disease Control and Prevention changed the term MDR *A. baumannii* to CR-Ab and included CR-Ab isolates among the “bacteria that urgently threaten the whole world” (4).

CR-Ab represents a significant cause of nosocomial infections (3). It can cause a number of infections, including pneumonia, bloodstream infections, urinary tract infections, soft tissue infections, meningitis, and endocarditis (2). Infections caused by CR-Ab are associated with a high incidence of morbidity and mortality (2). Treatment of serious CR-Ab infections is either by sulbactam-durlobactam or high-dose ampicillin-sulbactam, and colistin can be a combination agent (2, 3). Colistin is a polycationic peptide that is employed in clinical settings. Colistin, which was isolated from *Paenibacillus polymyxa* in 1947 and introduced in the 1950s, is a member of a group of antibiotics known as polymyxins (5). Colistin, which has not been used for a long time due to side effects such as nephrotoxicity and neurotoxicity, is used in combination therapy rather than monotherapy as the last treatment option (3).

Colistin resistance can develop through a number of different mechanisms, including the presence of multiple genes and operons. The *pmr*C, *pmr*E, and *pmr*HFIJKLM genes modify the structure of lipopolysaccharides, and regulatory systems such as PhoPQ and *pmr*A-*pmr*B oversee the PmrAB system. In addition to these mechanisms, plasmid-mediated *mcr* genes, *acr*B, *kpn*E, *cpx*A, and *cpx*R have been identified as contributing factors to colistin resistance (6).

The determination of susceptibility to colistin is a challenging process, primarily due to the complex nature of its macromolecular structure and the inherent difficulty in achieving adequate diffusion in agar (7). Therefore, the broth microdilution (BMD) method is currently the only reference method endorsed by both The European Committee on Antimicrobial Susceptibility Testing (EUCAST) and the Clinical and Laboratory Standards Institute (CLSI) (8). Despite its accuracy, BMD is labor-intensive and difficult to implement in routine diagnostic laboratories (9). Alternative methods have been developed to address this need, including the colistin broth disc elution method, which has also been endorsed by CLSI as an acceptable option for colistin MIC testing in clinical microbiology settings. In addition, commercial automated systems such as the VITEK 2 AST-XN21 card (bioMérieux, France) offer rapid and user-friendly platforms for susceptibility testing.

The present study investigates colistin susceptibility by employing both the BMD and the commercial VITEK 2 AST-XN21 (bioMérieux, Marcy l’Étoile, France) card. The automated system results were then compared with the gold standard BMD results. Furthermore, an in-house multiplex PCR method was utilized to investigate the presence of the potential resistance genes *mcr*-1, *mcr*-2, *mcr*-3, *mcr*-4, and *mcr*-5 in isolates exhibiting colistin resistance.

## MATERIALS AND METHODS

### Selection of isolates

A total of 187 CR-Ab isolates were obtained from the Clinical Microbiology Laboratory at Marmara University Pendik Training and Research Hospital between December 2020 and December 2022. Isolates were identified by MALDI-TOF MS and confirmed as carbapenem resistant using the Kirby-Bauer disk diffusion method. Duplicate isolates from the same patient were excluded from the study. All isolates were stored at −80°C in skim milk medium. In instances where there was a discrepancy between the BMD and VITEK 2 AST-XN21 results, the isolates were re-tested for BMD and VITEK 2 in order to confirm the findings.

### Antibiotic susceptibility testing

#### Broth microdilution method

The susceptibility of CR-Ab strains to colistin was investigated on polystyrene plates with a gold standard BMD across a concentration range of 64–0.125 mg/L, in accordance with ISO 20776 and EUCAST version 14 recommendations.

#### Determination of colistin susceptibility with VITEK 2 AST-XN21

The automated system used here to determine the MIC of colistin was the VITEK2 system. The cards used were VITEK2 AST XN-21 and processed following the manufacturer’s instructions (10). In summary, a suspension of 0.5 McFarland bacteria was prepared in 3 mL of saline. A volume of 145 μL of this homogeneous mixture was then taken with a pipette and transferred into 3 mL of saline solution, followed by homogenization. The resulting suspension was then loaded into the VITEK MS (BioMérieux, Marcy l’Étoile, France) automated system for evaluation. The cs02n version was used in the VITEK 2.

#### Quality control

*Escherichia coli* ATCC 25922 and *E. coli* NCTC 13846 were added, respectively, as negative (colistin susceptible) and positive (colistin resistant harboring *mcr-1*, MIC COL 4 mg/L) quality control strains for testing by BMD and VITEK 2. MIC was interpreted according to EUCAST breakpoints (susceptible ≤2 mg/L; resistant >2 mg/L) (11).

### Detection of resistance genes by PCR

As part of a supplementary analysis, isolates exhibiting phenotypic resistance to colistin were screened for the presence of *mcr* genes (*mcr*-1 to *mcr*-5) using a conventional PCR method.

#### DNA extraction

Isolates were suspended in 300 µL of sterile distilled water and centrifuged at 14,800 rpm for 5 minutes. After discarding the supernatant, the process was repeated once. The pellet was then resuspended in 500 µL of TE buffer (pH 8.0), followed by incubation at 98°C for 10 minutes. After a final centrifugation step (14,800 rpm for 5 minutes), the supernatant containing crude DNA was collected for PCR.

#### PCR protocol

PCR reactions were performed in a total volume of 25 µL using the GM SYBR qPCR Kit (Genemark), including 0.5 µM of each primer (Oligomer, Ankara), 1.5 mM MgCl_2_, 0.2 mM dNTP mix (Ampliqon), and 2 µL of DNA template. The primers used for the *mcr* gene detection are listed in Table 1.

**TABLE 1:** Primers used in our study

Primers	Sequence (5′−3′)	Amplicon size (bp)^*a*^
Mcr-1 F	CGGTCAGTCCGTTTGTTC	309
Mcr-1 R	CTTGGTCGGTCTGT GGG	
Mcr-2 F	TGTTGCTTGTGCCGATTGGA	567
Mcr-2 R	AGATGGTATTGTTGGTTGCTG	
Mcr-3 F	AAATAAAAATTGTTCCGCTTATG	929
Mcr-3 R	AATGGAGATCCCCGTTTTT	
Mcr-4 F	TCACTTTCATCACTGCGTTG	1,116
Mcr-4 R	TTGGTCCATGACTACCAATG	
Mcr-5 F	TGCGGTTGTCTGCATTTATC	1,644
Mcr-5 R	TCATTGTGGTTGTCCTTTTCTG	

^*a*^ bp, base pair.

Amplification was carried out in a T100 thermal cycler (Bio-Rad, USA) with the following steps: initial denaturation at 94°C for 15 minutes; 35 cycles of denaturation at 94°C for 30 seconds, annealing at 58°C for 90 seconds, and extension at 72°C for 60 seconds, followed by a final extension at 72°C for 10 minutes. PCR products were resolved on a 1.2% agarose gel stained with ethidium bromide and visualized under UV illumination following electrophoresis at 87 V for 35 minutes.

#### Quality control

*Escherichia coli* ATCC 25922 and *E. coli* NCTC 13846 (*mcr*-1 positive) were used as negative and positive controls, respectively.

### Statistical analysis

Isolates were evaluated for essential agreement (EA) and categorical agreement (CA), very major error (VME) rate, major error (ME) rate, and ISO 20776-2:2021 bias rate (acceptable if bias rate is between −30% ≤ bias ≤+30%.) (12).

## RESULTS

**Fig 1:**
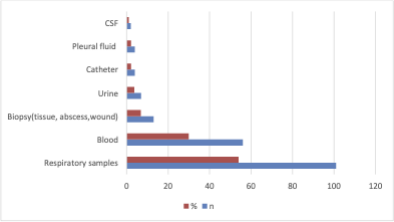
Distribution of CR-Ab isolates according to samples.

**Fig 2:**
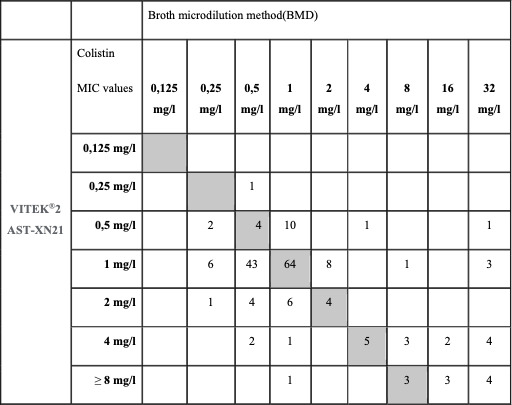
MIC distributions of all isolates determined by BMD and VITEK2AST-XN21.

**Fig 3:**
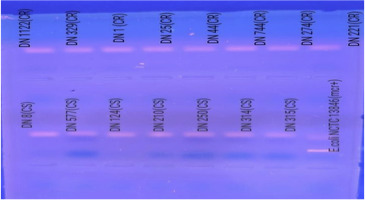
Gel electrophoresis of *mcr-1, mcr-2, mcr-3, mcr-4,* and *mcr-5* PCR products in colistin-resistant and susceptible clinical isolates. CR, colistin resistant; CS, colistin susceptible.

In this study, as shown in Fig. 2, out of the various specimens received, the highest number of CR-Ab isolates was found in respiratory specimens (54%, *n* = 110), followed by blood (29.9%, *n* = 56), biopsy specimens (6.9%, *n* = 13), urine (3.7%, *n* = 7), catheter (2.1%, *n* = 4), pleural fluid (2.1%, *n* = 4), and CSF (1.06%, *n* = 2) (Fig. 1). Out of these isolates, resistance to carbapenems (94.1%), aminoglycosides (95.1%), quinolones (95.1%), and piperacillin/tazobactam (95.1%) was detected by disc diffusion. Colistin resistance rate was found to be 16.04%. While the MIC_50_ in isolates is 1 mg/L, the MIC_90_ is 4 mg/L. When VITEK 2 AST-XN21 results were compared with the reference method in terms of colistin susceptibility, CA was found to be 94.6% (177/187), EA was 89.1% (156/175), and the bias rate was 3.6%. When MIC distributions were evaluated, VME was detected as 3.2% (6/187) and ME as 2.1% (4/187). BMD and colistin susceptibility distribution of the isolates studied with VITEK2 AST-XN21 are shown in Fig. 2. No *mcr-1*, *mcr-2*, *mcr-3*, *mcr-4,* and *mcr-5* genes were detected (Fig. 3).

## DISCUSSION

This study assessed the performance of the AST-XN21 VITEK 2 cards in determining colistin susceptibility, compared to the reference broth microdilution method. The findings of this study provide valuable insights into the utility of automated systems for colistin susceptibility testing in high-throughput clinical settings in a laboratory with a high volume of samples and a demanding workflow.

Among the CR-Ab isolates, 54% were obtained from respiratory tract samples and 29.9% from blood cultures. These findings are consistent with the well-documented role of CR-Ab in ventilator-associated pneumonia and bloodstream infections in critically ill patients (13–16).

In our study, colistin resistance was found to be 16.04% in *A. baumannii* isolates by BMD. When the studies on colistin resistance are examined, Maraki et al. (17) reported a resistance rate of 7.9%, Gao et al. (18) reported 3%, and in the meta-analysis conducted by Lima et al. (19), colistin resistance was found to be 13% in *A. baumannii* isolated from inpatients. In another meta-analysis, colistin resistance has increased over the years, and resistance ranging from 1% to 60% has been reported in different parts of the world (20).

When the performance of the automated systems was analyzed for colistin, some studies reported VITEK 2 testing to be reliable, others were unable to reproduce these findings. Vourli et al. (21) compared the performance of the automated Phoenix100, VITEK 2, and agar dilution methods with the reference method BMD systems in a study involving 117 CR-Ab isolates. They detected 24.8% of colistin resistance in CR-Ab isolates with BMD. They found that CA was 89.7%, EA was 88.9%, and VME was 37.9% with the VITEK 2 system. They reported that isolates found susceptible with the automated system should be confirmed with a reference method. It was also stated that there is a need for a new rapid method that can be used in routine laboratories. The manufacturer states that the method is not recommended for *Pseudomonas* spp. but may be a reliable alternative for Enterobacterales and *A. baumannii* isolates (10). In other studies conducted with the VITEK 2 (used cs01n) system in the literature, colistin susceptibility was tested with the reference method. There are studies indicating that the results of the automated system are reliable in determining colistin susceptibility in Enterobacterales and *A. baumannii* isolates (22, 23). However, there are also publications stating that the error rates are high and incompatible results are obtained with the reference method, so it is not recommended (24–26). In another study with 213 *A*. *baumannii* isolates, 38% (81/213) colistin resistance was detected with the reference method, BMD. In addition, the performance of the Vitek automated system was investigated in the study. CA was 95.3%, VME was 9.9%, and they reported that the Vitek system was not acceptable in determining colistin susceptibility (27). Ananda et al. (28) studied 284 Enterobacterales with a total of 533 gram-negative bacteria. They tested colistin susceptibility of Enterobacterales isolates with AST-N280 and the colistin susceptibility of non-fermenter strains with AST-N281. They reported VITEK 2 performance with a sensitivity of >99%, specificity of 14.28%, VME of 52.94%, ME of 20%, EA of 68.5%, and CA of 99.79%.

In our study with XN21, which contains the latest version of the drug (cs02n), the VITEK 2 XN21 card showed >90% CA and 3.2% (6/187) VME rates compared to the reference method, BMD. This result is very promising, especially considering that VITEK 2 is one of the most widely used automated systems in routine laboratories.

In our study, the *mcr-1* to *5* genes, one of the gene regions responsible for colistin resistance, were investigated by an in-house PCR method, and the *mcr* genes were not detected. We attribute this to the fact that other mechanisms related to colistin resistance were effective in our isolates. In studies investigating the relationship between colistin resistance and *mcr* in various microorganisms, many studies did not detect *mcr* in *Acinetobacter* species (29–31).

### Conclusion

The VITEK2 AST-XN21 card demonstrates a promising performance for colistin susceptibility testing, with >90% CA and a relatively low VME rate (3.2%) compared to the reference BMD method. Given its rapid turnaround time and practicality, it represents a valuable tool for routine laboratory workflows. However, due to the critical nature of accurate MIC determination in managing CR-Ab infections, especially in severe clinical cases, reliance solely on automated systems may not suffice. Confirmatory testing with BMD remains essential in such scenarios.
